# Improvements in Cd stable isotope analysis achieved through use of liquid–liquid extraction to remove organic residues from Cd separates obtained by extraction chromatography†
†Electronic supplementary information (ESI) available. See DOI: 10.1039/c5ja00115c



**DOI:** 10.1039/c5ja00115c

**Published:** 2015-08-18

**Authors:** Katy Murphy, Mark Rehkämper, Katharina Kreissig, Barry Coles, Tina van de Flierdt

**Affiliations:** a Department of Earth Science and Engineering , Imperial College London , London SW7 2AZ , UK . Email: k.murphy12@imperial.ac.uk ; Tel: +44 (0)2075947140

## Abstract

Organic compounds released from resins that are commonly employed for trace element separations are known to have a detrimental impact on the quality of isotopic analyses by MC-ICP-MS. A recent study highlighted that such effects can be particularly problematic for Cd stable isotope measurements (M. Gault-Ringold and C. H. Stirling, *J. Anal. At. Spectrom.*, 2012, **27**, 449–459). In this case, the final stage of sample purification commonly applies extraction chromatography with Eichrom TRU resin, which employs particles coated with octylphenyl-*N*,*N*-di-isobutyl carbamoylphosphine oxide (CMPO) dissolved in tri-*n*-butyl phosphate (TBP). During chromatography, it appears that some of these compounds are eluted alongside Cd and cannot be removed by evaporation due to their high boiling points. When aliquots of the zero-ε reference material were processed through the purification procedure, refluxed in concentrated HNO_3_ and analyzed at minimum dilution (in 1 ml 0.1 M HNO_3_), they yielded Cd isotopic compositions (ε^114/110^Cd = 4.6 ± 3.4, 2SD, *n* = 4) that differed significantly from the expected value, despite the use of a double spike technique to correct for instrumental mass fractionation. This result was accompanied by a 35% reduction in instrumental sensitivity for Cd. With increasing dilution of the organic resin residue, both of these effects are reduced and they are insignificant when the eluted Cd is dissolved in ≥3 ml 0.1 M HNO_3_. Our results, furthermore, indicate that the isotopic artefacts are most likely related to anomalous mass bias behavior. Previous studies have shown that perchloric acid can be effective at avoiding such effects (Gault-Ringold and Stirling, 2012; K. C. Crocket, M. Lambelet, T. van de Flierdt, M. Rehkämper and L. F. Robinson, *Chem. Geol.*, 2014, **374–375**, 128–140), presumably by oxidizing the resin-derived organics, but there are numerous disadvantages to its use. Here we show that liquid–liquid extraction with *n*-heptane removes the organic compounds, dramatically improving quality of the Cd isotope data for samples that are analyzed at or close to minimum dilution factors. This technique is quick, simple and may be of use prior to analysis of other isotope systems where similar resins are employed.

## Abstract 2

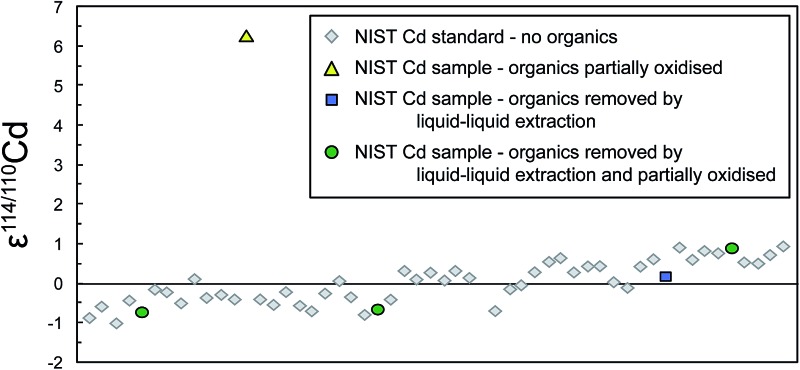
During isotopic analysis of Cd by MC-ICP-MS, organic resin residue can perturb instrumental mass fractionation. These organic compounds can be removed by a liquid–liquid extraction procedure using heptane.

## 1. Introduction

Recently, interest in the measurement of Cd isotopes has grown due to advances in mass spectrometry and the realization that there are resolvable isotopic differences in seawater, meteorites and other samples of geological, biological and anthropogenic origin. As natural terrestrial samples display only small isotopic differences, Cd isotopic compositions are most commonly reported using an ε notation as follows:1
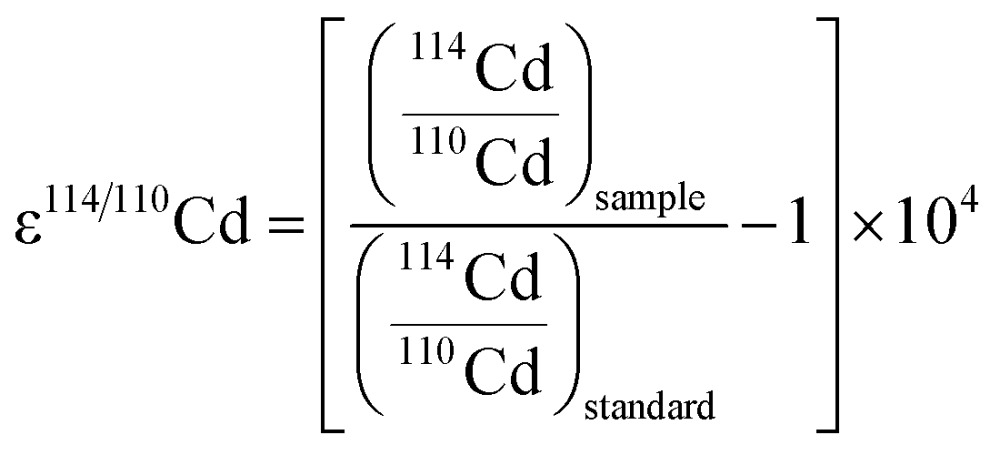
where the standard reference material most widely adopted (and used here) is NIST SRM 3108 Cd.^1^ Isotopic compositions with ε^114/110^Cd values of between +50 and –7 (for surface seawater from off north west Africa,^2^ and for North Pacific surface water, respectively^3^) have been found in the natural terrestrial environment. Even larger fractionations can be produced by processes that involve partial evaporation and condensation of Cd, and the signatures of such reactions are found in, for example, materials from industrial processes (ε^114/110^Cd = –17 to +5 (ref. 4–6)) and meteorites (–80 to +160 (ref. 7)).

The dominant method for the determination of Cd isotope compositions applies multiple collector inductively coupled plasma mass spectrometry (MC-ICP-MS), with instrumental mass bias correction by the double spike technique.^8,9^ Prior to the measurements, Cd is separated from the sample matrix by a first stage of anion exchange chromatography, followed by extraction chromatography with Eichrom TRU resin for the removal of Sn.^8–15^

Various Eichrom resins, as well as similar homemade resin materials, are commonly employed for extraction chromatography in geochemical laboratories for the purification of numerous trace metals. Amongst users, it is common knowledge that the organic extraction agent (present as a coating on the resin particles) to some extent always elutes alongside the analyte element. This organic material can have a detrimental impact on isotopic analyses by TIMS (thermal ionization mass spectrometry) if it hinders the ionization process.^16^ For MC-ICP-MS, the presence of the organic residue appears to be relatively unproblematic for many analyses. In such cases, any effects caused by the organic material are presumably sufficiently small to be of no concern and this is supported by minimizing the amount of residue present, through pre-leaching the coated particles by storage in water or dilute acids and/or cleaning of the resin within the columns by acid elution directly prior to use.^11,17^

In some cases, however, problems can occur despite such precautions. In particular, Gault-Ringold and Stirling^13^ reported that the Cd isotope data for selected samples displayed a reproducibility and accuracy that was poor compared to that achievable for unprocessed standard solutions and concluded that this was caused by the organic residue that eluted alongside Cd from the TRU resin that was used in their chromatographic procedure. They furthermore suggested that this problem can be avoided if sufficiently large samples are processed on a single column. In this case, the final Cd sample solutions for isotopic analysis are diluted to a relatively large volume such that the eluted organic material is not present in concentrated form.^13^ In practice, however, this is often not possible. The concentration of Cd in seawater can be as low as 0.1 pg g^–1^ (or 1 pmol l^–1^), whilst other samples, such as meteorites, mineral separates or ferromanganese crusts, may only be available in small quantities.^18^

In cases where resin pre-rinsing or sample dilution is unable to circumvent the analytical problems that can be caused by organic resin residues, the material must be separated from the target element after extraction chromatography. To improve the quality of Cd and Nd isotope analyses following use of Eichrom resins, it was suggested that the organic material is best removed by oxidation of the residue with reagents such as HNO_3_, HNO_3_ with added H_2_O_2_, or HClO_4_.^13,19,20^ Of these, HClO_4_ was found to be the most effective oxidant,^13,20^ but there are many disadvantages to its use, such as various safety considerations, a high boiling temperature, and the availability of sufficiently pure supplies. Oxidation with HNO_3_ + H_2_O_2_, can also be successful but is more time consuming (24 hours).^13^ In the current study, we have developed an improved alternative method for the removal of organic resin residues from Cd sample solutions for subsequent isotopic analysis by MC-ICP-MS. The procedure avoids use of oxidising agents entirely, as the organic compounds are separated by liquid–liquid extraction with heptane, and may be applicable for improving the sample preparation procedures of other elements prior to isotopic measurements.

## 2. Method

## 2.1 Materials and reagents

All sample preparation work was carried out in class 10 (ISO 4) laminar flow hoods in the class 1000 (ISO 6) MAGIC clean room laboratory at the Department of Earth Science and Engineering, Imperial College London. Some acids were purified by sub-boiling distillation in either Teflon (12 M HCl) or quartz (6 M HCl, 16 M HNO_3_) stills, whilst Optima grade 9 M HBr was purchased from Fisher Scientific. The HBr–HNO_3_ mixtures were prepared on the day of use and all water was of 18.2 MΩ quality from a Milli-Q Academic dispensing system. The ^111^Cd–^113^Cd double spike, with ^113^Cd/^111^Cd = 0.5829, in 2 M HCl was prepared from solutions of enriched single isotopes purchased from Oak Ridge National Laboratory (USA) and characterized by MC-ICP-MS using external normalization relative to admixed Ag.^8^

Heptane was pre-cleaned by carrying out a liquid–liquid extraction with 6 M HCl. Specifically, approximately 40 ml of Alfa Aesar 99% *n*-heptane was shaken by hand for 30 seconds with approximately 20 ml 6 M HCl in a 90 ml Savillex beaker and then left to stand for approximately 3 minutes. Once phase separation had been verified, the beaker was shaken again and allowed to stand for a further three minutes. The heptane was then transferred to a clean 90 ml Savillex beaker, being careful not to take up any of the underlying acid. This process was repeated twice for further purification.

## 2.2 Samples

Two different samples were used. The first, a pure solution of NIST SRM 3108 Cd (hereafter also referred to as NIST Cd), was employed so that complications arising from matrix effects due to the presence of other elements were avoided. Direct comparison between processed samples and unprocessed NIST Cd was enabled by using NIST Cd as the ε^114/110^Cd = 0 reference material to bracket the sample analyses. A second sample, the powdered ferromanganese nodule reference material Nod-A-1 from the USGS, was chosen as a natural material that is readily available. The Cd isotope composition of this material has been characterized previously as ε^114/110^Cd = 2.3 ± 0.6 (2SE, *n* = 2 (ref. 11)) and ε^114/110^Cd = 1.3 ± 0.2 (2SE, *n* = 2 (ref. 21)), possibly reflecting minor sample heterogeneity.

## 2.3 Sample preparation

Aliquots of Nod-A-1 were digested and purified following the procedure described by Horner *et al.* ^11^ In brief, this involved digestion of the sample powder with 6 M HCl, addition of double spike to obtain an optimum ratio of spike Cd/natural Cd of S/N ≈ 1,^9^ anion exchange chromatography with 200 μl Bio-Rad AG1-X8 resin (200–400 mesh size; for separation of Cd from the matrix) and then extraction chromatography with 200 μl Eichrom TRU resin (100–150 mesh size; primarily to remove any remaining Sn). The Eichrom TRU resin was pre-cleaned by shaking with 18.2 MΩ water and removing the foam that was produced. This was repeated 5–10 times (until no more foam appeared) and then stored in 18.2 MΩ water. Once settled in the columns the resin was cleaned with 9 ml 6 M HCl.^11^ As the NIST Cd solution is sufficiently pure to make the first stage of column chemistry redundant, samples using this solution were only processed through the second stage of the separation procedure before any attempts were made to remove the organic resin residue.

## 2.4 Organic resin residue removal

At this point, the samples were further treated using one of four different protocols and therefore we differentiate between *untreated*, *refluxed*, and *extracted* samples, and samples that were both *extracted* and *refluxed* or *refluxed* and *extracted* (note the different order). Unless otherwise specified, each individual sample processed separately through the column chemistry contained approximately 30 ng of natural Cd plus 30 ng Cd from the double spike for a total of 60 ng Cd. Following the treatments described in Table 1, most samples were dissolved in 1 ml 0.1 M HNO_3_ to produce a 60 ng ml^–1^ solution for analysis by MC-ICP-MS. This approach highlights the problem caused by organic compounds eluted from the TRU resin, as it simulates the scenario where the Cd content of a sample suffices only for a single analysis (a ‘one shot’ sample), so that dilution of the organics is not possible.

**Table 1:** Summary of organic residue removal methods

Sample treatment protocol	Elution dried down	Dried with 1 drop 16 M HNO_3_	Refluxed^*a*^	Dried down	Dissolved in 1 ml 0.1 M HNO_3_	Extraction procedure^*b*^	Refluxed^*a*^	Dried down	Dissolved in enough 0.1 M HNO_3_ to produce a 60 ng ml^–1^ solution
Untreated	✓	✓✓							✓^*c*^
Refluxed	✓	✓	✓					✓	✓^*d*^
Extracted						✓		✓	✓^*c*^
Extracted and refluxed						✓	✓	✓	✓^*c*^
Refluxed and extracted	✓	✓	✓	✓	✓	✓		✓	✓^*c*^

^*a*^Refluxed in 0.5 ml 16 M HNO_3_ at 140 °C for 5 days.

^*b*^Extraction procedure is as follows: 1.2 ml pre-cleaned *n*-heptane added to beaker. Shaken for 30 seconds, stood for 3 minutes, repeated once. Organic upper layer carefully removed with a pipette. 1.2 ml pre-cleaned *n*-heptane added. Shaken for 30 seconds, stood for 3 minutes, repeated once. Organic upper layer carefully removed with a pipette. Residual heptane allowed to evaporate at ambient temperature in a laminar flow hood for 30–60 minutes.

^*c*^1 ml 0.1 M HNO_3_.

^*d*^Most refluxed samples contained 60 ng total Cd, but some had up to 240 ng. All samples, however, had S/N ≈ 1 and were dissolved in the appropriate amount of 0.1 M HNO_3_ to produce solutions with a total Cd concentration of 60 ng ml^–1^ for analysis. In one exceptional case, two samples, each with 30 ng total Cd dissolved in 0.5 ml 0.1 M HNO_3_, were combined to produce a single 1 ml solution with 60 ng ml^–1^ Cd. This approach enabled us to generate Cd solutions that featured variable dilution factors for the eluted organic resin compounds.

## 2.5 Mass spectrometry

All samples were analyzed on a Nu Plasma HR MC-ICP-MS (Nu Instruments Ltd, Wrexam, UK) at the MAGIC Laboratories. For sample introduction, either an Aridus I or Aridus II desolvating nebulizer system was used, fitted with a PFA nebulizer (CETAC Technologies) operating at a solution flow rate of about 120 μl min^–1^. The data acquisition procedures were similar to those outlined by Xue *et al.* ^9^

Solutions of spiked NIST Cd with S/N ≈ 1 and Cd concentrations similar to those of samples were repeatedly analyzed as bracketing standards throughout each measurement session, with between three and eight standards run between each sample. This allowed the stability of the instrument to be monitored, and any change in mass bias to be observed. Each analysis typically consumed about 30 ng natural Cd and 30 ng spike Cd, and a sensitivity of between 200 and 300 V (μg ml^–1^)^–1^ was normally achieved during the course of the study. All runs were bracketed by approximately four minutes of rinsing with 0.1 M HNO_3_.

The ε^114/110^Cd values of the samples were determined offline, by using the online collected raw ion beam intensities and electronic baselines as input to an Excel spreadsheet. The spreadsheet applies previously outlined iterative methods to correct the measured ^112^Cd/^111^Cd, ^113^Cd/^111^Cd, and ^114^Cd/^111^Cd isotope ratios for isobaric interferences and solve the double spike equations.^8,9,22^ The general power law is applied using a mass dependence of *n* = –0.1 to correct for instrumental mass fractionation.^23^

Calculations analogous to eqn (1) were performed to obtain the ε^114/113^Cd values of samples, relative to the results that were obtained for bracketing analyses of spiked solutions of NIST SRM 3108 Cd. In the majority of cases, the three or four analyses of the standard solution on either side of the sample were chosen. On occasions where the instrument was particularly stable, many more (up to 68) analyses of the standard solution were included.

Finally, the ε^114/113^Cd data were translated into ε^114/110^Cd using the relationship2
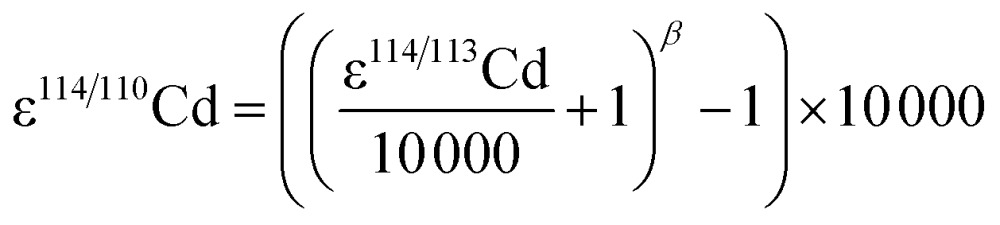



The exponent, *β*, was determined assuming the kinetic law3
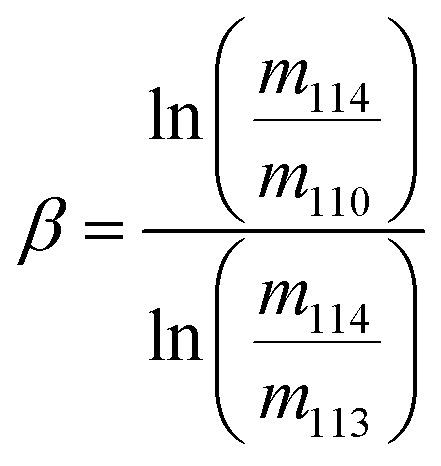
where *m*_*i*_ is the atomic mass of isotope *i*.

Samples were excluded from the data set where either the internal (within-run) 2SE or the external (bracketing standards) 2SD uncertainties exceeded ±1 ε^114/110^Cd, except for occasional cases where the internal 2SE (typically better than ±0.8*ε*) was not unusually high for the measurement session. As unstable instrument behavior is problematic, Table 2 also highlights the number of sample measurements that were discarded relative to the total number of attempted analyses for the different sample treatments that were investigated. Such data selection was necessary to enable a reasonable evaluation of the results, as is further explained in the discussion below. The complete data set, including all discarded measurements, is provided in the ESI.†

**Table 2:** Mean Cd isotope composition of samples with approximately 60 ng total Cd and analyzed at 60 ng ml^–1^ Cd. All individual data are provided in the ESI

Sample	Source	Treatment after purification	Mean ε^114/110^Cd	2SD	Number analyses included	Number analyses discarded^*a*^
NIST SRM 3108 Cd^*b*^	This study	None	–0.1	0.8	6	1
	This study	Refluxed	4.6	3.4	4	4
	This study	Extracted	0.0	0.5	6	2
	This study	Extracted and refluxed	0.0	0.7	9	1
	This study	Refluxed and extracted	3.0	0.8	3	0
USGS SRM Nod-A-1^*c*^	This study	None	2.5	3.4	2	1
	This study	Refluxed	4.3	5.9	2	0
	This study	Extracted (preferred value)	1.7	0.5	6	0
	This study	Extracted and refluxed	2.1	0.7	2	0
	This study	Refluxed and extracted	3.6	1.2	2	0
	Horner *et al.* ^11^	None	2.3	0.6^*d*^	2	
	Schmitt *et al.* ^21^	None^*e*^	1.3	0.2	2	
BAM-I012	This study	No purification procedure	–13.2	0.7	15	0
	Abouchami *et al.* ^1^	No purification procedure	–13.3	0.4		

^*a*^Analyses were excluded when either the internal precision (2SE) or the external precision (2SD) exceeded ±1 ε^114/110^Cd, unless the internal 2SE was not unusual for that particular instrument session (see text for details).

^*b*^NIST Cd samples underwent just the second stage of the separation chemistry.

^*c*^Nod-A-1 samples underwent both stages of the separation chemistry.

^*d*^Reported uncertainty is 2SE, not 2SD.

^*e*^Analysed by thermal ionisation mass spectrometry (TIMS).

Changes in the instrumental mass bias of Cd during measurements sessions were determined by monitoring changes in the ^111^Cd/^114^Cd ratio of the spike-sample mixtures and quantified in terms of the fractionation coefficient (sometimes also called beta factor, mass bias factor or fractionation factor), *f*_Kin_ which is applied in the exponential mass fractionation law:4
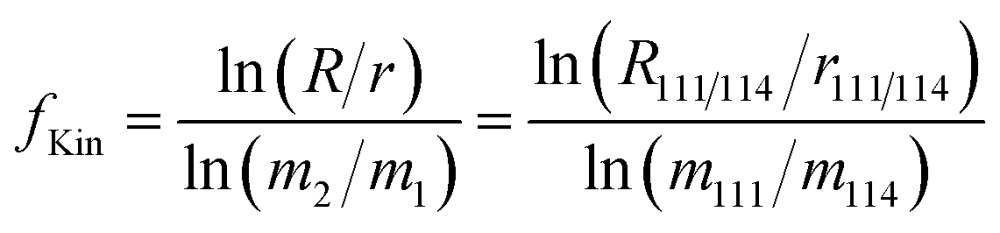
where *R* is the unbiased or ‘assumed true’ isotope ratio corrected for instrumental mass bias, *r* is the same isotope ratio but fractionated relative to the true value due to instrumental mass bias, and *m*_*i*_ is the atomic mass of isotope *i*.^23^

Typical procedural blanks contained <20 pg Cd. As the blank constitutes just 0.03% or less of the total Cd in a sample, no blank corrections were applied to the isotope compositions. Repeat measurements of the secondary Cd isotope standard BAM-I012 gave ε^114/110^Cd = –13.2 ± 0.7 (2SD, *n* = 15), which is in excellent agreement with the consensus literature value of –13.3 ± 0.4.^1^

In the following discussion, the quoted uncertainties for mean values refer to twice the standard deviation (2SD) of the individual sample results included in this average. The uncertainty of individual results refers to twice the standard deviation (2SD) obtained for bracketing runs of the NIST Cd standard.

## 3. Results and discussion

### 3.1 Isotope ratios and sensitivity

#### 3.1.1 Untreated samples

For all untreated samples, a drop of transparent residue of about 2.5 mm remained after drying down the collected column elution. The analyses of such samples were associated with variable mass bias behavior and variable results. This is one of the features of handling samples in this manner and is an indicator that something is disturbing the behavior of the machine.

**Fig. 1:**
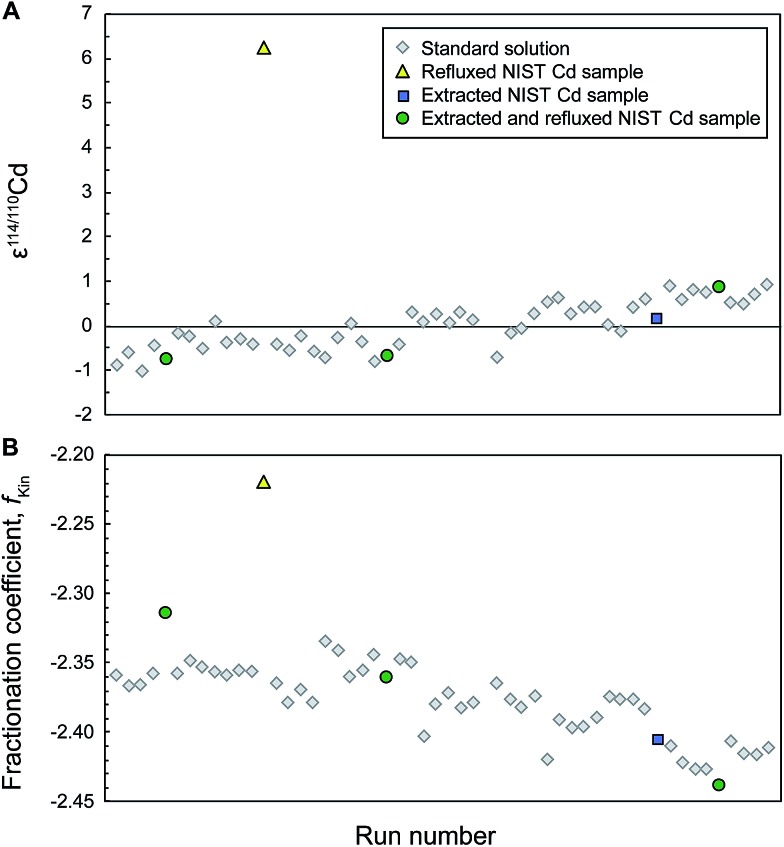
Typical analytical session of repeat measurements of the NIST SRM 3108 Cd standard solution doped with the Cd double spike (to S/N ≈ 1) together with interspersed measurements of aliquots of NIST SRM 3108 Cd that were passed through the extraction chromatography separation stage (Eichrom TRU resin) and subsequently extracted and/or refluxed to remove the organic residue. All solutions and standards should have identical isotope composition as they all contain only (spiked) NIST Cd, yet the refluxed sample is offset by 6*ε* (A). Simultaneous shifts in the instrumental fractionation coefficient *f*_Kin_ are shown in (B).

Analyses of NIST Cd samples all gave the correct isotope composition and a good overall reproducibility (ε^114/110^Cd = –0.1 ± 0.8, *n* = 6; Table 2). There was also no disruption to the isotope composition of following standards, but the observation of sudden shifts in *f*_Kin_ of up to 0.07 (Fig. 1) suggests that there are mass bias changes between samples and subsequent standard analyses.

The results for the Nod-A-1 samples also provide a mean that is in accord with reference data (Table 2) but the overall precision is unsatisfactory (ε^114/110^Cd = 2.5 ± 3.4, *n* = 2). Further details of these analyses can be found in the ESI.†

#### 3.1.2 Refluxed samples

Samples that were refluxed in concentrated HNO_3_ following extraction chromatography displayed isotope compositions that were heavier and more variable than expected. In particular, the NIST Cd samples provide a mean ε^114/110^Cd of +4.6 ± 3.4 (Table 2), which deviates significantly from the expected value (of ε^114/110^Cd = 0) and that shows a very poor reproducibility. Similarly, the two refluxed Nod-A-1 samples also reproduce poorly with ε^114/110^Cd values of +6.4 ± 0.6 and +2.2 ± 0.7. Clearly, this method of breaking down the organic resin residue is ineffective, and may in fact amplify the problem.

We also observed that many runs of refluxed NIST Cd clearly disturbed the instrumental running conditions, so that subsequent measurements of the bracketing NIST Cd standard yielded isotope compositions that were substantially offset from the runs immediately preceding the sample analysis. Such effects are most likely due to the presence of organic resin residue in the refluxed NIST Cd samples. In detail, the isotopic offset between the bracketing standard measurements was large enough, such that they displayed 2SD precisions exceeding ±1*ε* for about 50% of the refluxed NIST Cd samples (Table 2). In these cases, it was not possible to robustly calculate the ε^114/110^Cd values of the samples relative to the bracketing standards, and these measurements were, therefore, not included in calculation of the mean given in Table 2. The rational for this approach is that such analyses would also be recognized as problematic and discarded when ‘unknowns’ are analyzed.

**Fig. 2:**
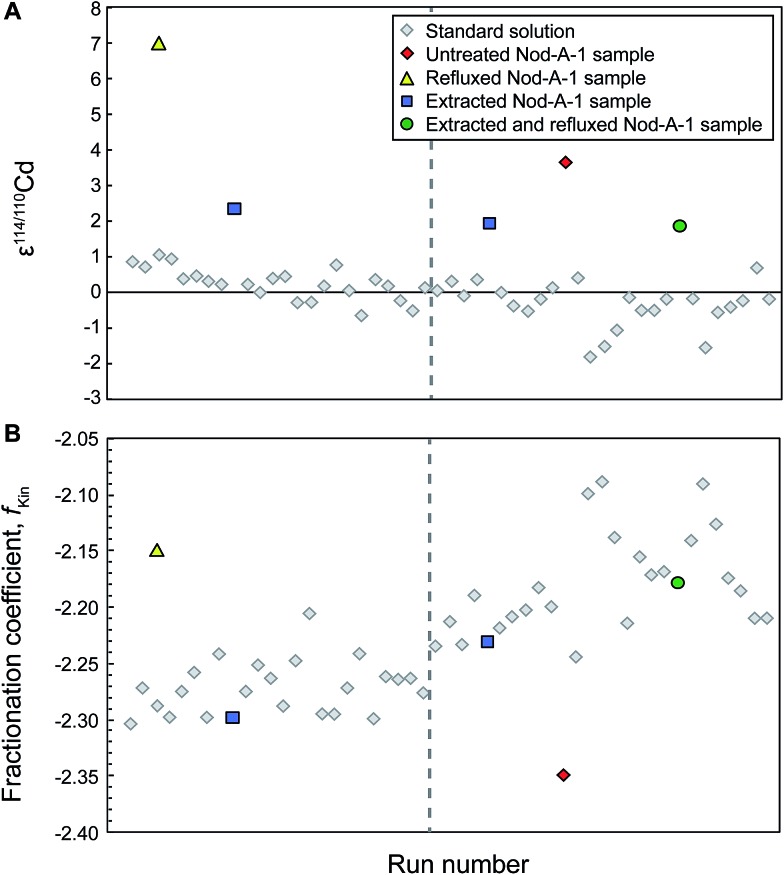
Typical analytical session of repeat measurements of the NIST SRM 3108 Cd standard solution doped with Cd double spike together with interspersed measurements of the ferromanganese nodule Nod-A-1 that passed through both stages of column chemistry and were subsequently extracted and/or refluxed to remove the organic residue, or left untreated. The dashed grey line indicates when the sweep gas of the desolvating sample introduction system was adjusted; this typically leads to a change in the instrumental mass bias. All samples of Nod-A-1 should yield identical isotope compositions (A). For this particular instrument session, samples that were cleaned by extraction or extraction and refluxing give ε^114/110^Cd = 2.1 ± 0.4 (2SD), whilst the untreated and particularly the refluxed samples have much higher ε^114/110^Cd values. Note the delayed 2*ε* shift in the isotope data for the NIST Cd standards that were analyzed following the untreated sample. The shift occurs only with the second standard analyses after the sample and the instrumental fractionation coefficient *f*_Kin_ does not display a simple step change after this analysis (B).

Accompanying these unexpected Cd isotope compositions, we also observed that the fractionation coefficient *f*_Kin_ for the refluxed NIST Cd samples frequently (for 5 out of 8 sample analyses) differed substantially from the bracketing standards (Fig. 1). This was also the case for the refluxed sample of Nod-A-1 shown in Fig. 2, indicating that the mass bias behavior is different for refluxed samples and pure, unprocessed NIST Cd solutions.

**Fig. 3:**
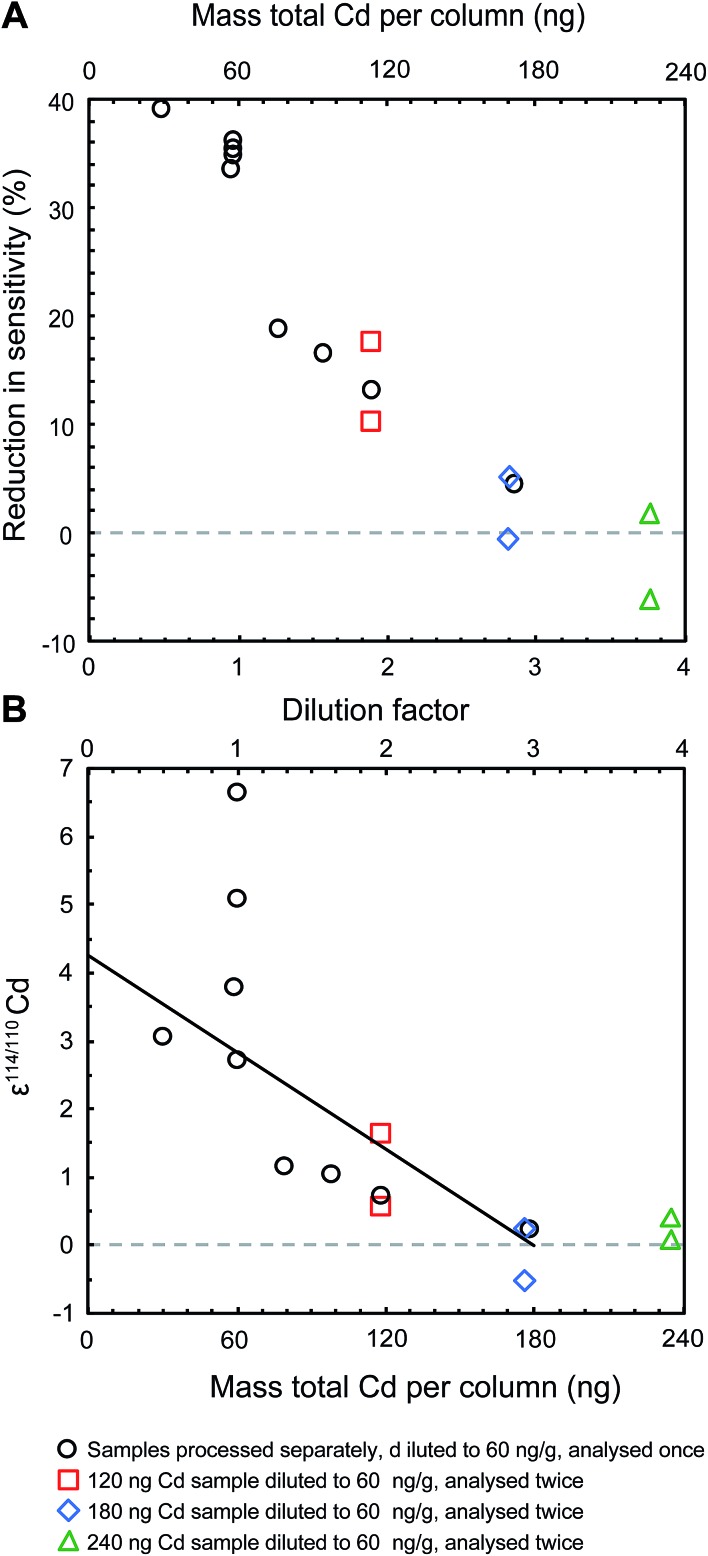
The presence of an organic residue reduces the sensitivity of the instrument (A) and produces analyses that yield inaccurate ε^114/110^Cd data (B). All samples shown are aliquots of the NIST SRM 3108 Cd standard solution doped with the Cd double spike. They were passed through the second stage of the column chemistry that applies Eichrom TRU resin, subsequently refluxed in concentrated nitric acid and then diluted to 60 ng ml^–1^. The solid trend line in panel (B) is shown to illustrate that the changes in ε^114/110^Cd with dilution factor are not appropriately described by a simple linear trend.

The analyses of refluxed samples also revealed that erroneous Cd isotope compositions were consistently accompanied by a reduced instrumental sensitivity for Cd (Fig. 3). Refluxed NIST Cd samples with a total Cd content of 60 ng, had been processed by extraction chromatography (Table 2), refluxing with 16 M HNO_3_ and dilution with 0.1 M HNO_3_ to a Cd concentration of about 60 ng ml^–1^. Such samples produced an instrumental sensitivity for Cd that was up to almost 40% lower, compared to the Cd sensitivity recorded by the unprocessed bracketing standards (Fig. 3). When larger Cd samples were refluxed and diluted to larger volumes, thereby producing more favourable (larger) ratios of Cd to organic residue, the sensitivity improved and the measured isotope compositions were closer to the expected value of ε^114/110^Cd = 0. Results that were unbiased by the presence of organic resin residue were achieved when samples with at least 180 ng total Cd were processed and diluted to a Cd concentration of 60 ng ml^–1^ using at least 3 ml 0.1 M HNO_3_ (*i.e.* a dilution factor of three; Fig. 3).

#### 3.1.3 Extracted samples

After drying down the Cd fraction following column chemistry and extraction, the remaining residue, typically a nearly transparent drop with a diameter of ∼0.5 mm, was noticeably smaller compared to that left by untreated samples (where the diameter of the drop was about 2.5 mm). Furthermore, all analyses of extracted samples showed the expected isotope compositions within uncertainty and replicate measurements displayed excellent reproducibility. Analyses of extracted NIST Cd yielded a mean ε^114/110^Cd = 0.0 ± 0.5 (Table 2), whereby the reproducibility is essentially identical to that achieved for multiple measurements of the untreated BAM I012 Cd reference material (Table 2) and replicate analyses of unprocessed NIST Cd standards within a single measurement session. The measurements of extracted Nod-A-1 samples were of similar quality, again yielding a precise mean of ε^114/110^Cd = 1.7 ± 0.5, which is identical to reference results (Table 2). It is also encouraging that the fractionation coefficient *f*_Kin_ observed for analyses of extracted samples is substantially closer to that of bracketing standard measurements in comparison to untreated or refluxed samples (Fig. 1 and 2). This implies that extracted samples typically ‘behave’ in the mass spectrometer in a nearly identical fashion to completely unprocessed pure Cd standard solutions. Furthermore, none of the six extracted Nod-A-1 samples and just two of the eight extracted NIST Cd analyses were excluded from the calculations due to unfavourable internal or external uncertainties. The poor repeatabilities recorded by the two NIST Cd measurements most likely reflect disturbances in the mass bias behavior from previous analyses of untreated and refluxed samples that were carried out in the same measurement session (see ESI†).

The total blank for the procedure including the extraction was only about 10 pg Cd, and as this was less than 0.02% of the total Cd for most samples, no correction was applied to ε^114/110^Cd data. The low blank was not unexpected given that cleaned *n*-heptane was found to have undetectable levels of Cd.

Our results, therefore, indicate that the extraction method can provide near complete removal of the organic material from the aqueous phase. Our method is, furthermore, easy to implement, as no unusual reagents or instrumentation is required, and was found to be reliable on a routine basis. In addition, it is quick in its use, as a batch of 10 samples can be readily extracted in about 30 minutes.

#### 3.1.4 Extracted and refluxed samples

The results for Cd samples that were extracted and then refluxed showed the expected isotope composition and good reproducibility (Table 2), both for NIST Cd (mean ε^114/110^Cd = 0.0 ± 0.7, *n* = 9, one excluded analysis) and for two aliquots of Nod-A-1 (ε^114/110^Cd = 2.0 ± 1.2 and 2.4 ± 0.9, no excluded analyses). In addition, the *f*_Kin_ values obtained for these samples were similar to the bracketing standards. However, no significant improvement is seen compared to carrying out the extraction procedure only, suggesting that the additional refluxing step is unnecessary.

#### 3.1.5 Refluxed and extracted samples

Analyses of refluxed and extracted samples show isotope compositions that are isotopically heavier than expected, with mean data of ε^114/110^Cd = 3.0 ± 0.8 (*n* = 3) for NIST Cd and ε^114/110^Cd = 3.6 ± 1.2 (*n* = 2) for Nod-A-1 (Table 2). These poor results most likely reflect partial oxidation of the organic resin residues to secondary compounds that are more soluble in water, and which are therefore not sufficiently removed from the aqueous phase by liquid–liquid extraction.

### 3.2 Causes of poor analytical results

Most analyses of untreated and refluxed samples yielded inaccurate data combined with poor reproducibility. These effects are inferred to result from contamination of the Cd sample solutions by organic compounds, which are released from the Eichrom TRU resin that is used for purification. Eichrom TRU resin applies octylphenyl-*N*,*N*-di-isobutyl carbamoylphosphine oxide (CMPO) dissolved in tri-*n*-butyl phosphate (TBP)^24^ as active ingredients. These long chain organic compounds have boiling points of more than 480 °C (ref. 25) and hence they are not removed by evaporation from Teflon beakers, and oxidation with concentrated nitric acid is insufficient.

Our findings are in general accord with the study of Gault-Ringold and Stirling.^13^ These workers found large shifts in the Cd isotope composition of bracketing standards after samples containing organic resin residue had been analysed. Gault-Ringold and Stirling^13^ also applied Cd purification by extraction chromatography with TRU resin and the mass spectrometric methods, including double spiking, were similar to those used here. To circumvent this problem, the authors suggested that samples should be oxidized with HClO_4_/HNO_3_ or H_2_O_2_/HNO_3_ mixtures and they show that samples treated in this manner yield accurate and precise results. This method appears to be successful because the treatment with such media likely oxidizes the majority of the organic material present, possibly largely to CO_2_ and H_2_O. Crocket *et al.*,^20^ who use Eichrom RE (CMPO dissolved in TBP but at a different ratio to that of TRU resin) and Ln resins to separate neodymium in fossil corals, found that organic compounds from the RE resin had a detrimental effect on Nd yields and that oxidation of the sample aliquot after the RE chemistry using HClO_4_ solved the problem. However, this approach can be further improved. Oxidation of the organic residue may be incomplete in some cases, and this is difficult to monitor. At the very least, even complete oxidation of CMPO and TBP will leave behind a small residue of H_3_PO_4_, which cannot be effectively removed from Teflon beakers by evaporation due to its high boiling point of 213 °C.

Gault-Ringold and Stirling^13^ concluded that the analytical artefacts stemming from the use of TRU resins were caused either by polyatomic interferences (*e.g.*, organophosphorus compounds) or anomalous mass bias behavior, such as mass independent or non-exponential mass dependent isotope fractionation. The results of modelling that was carried out to study the impact of spectral interferences show (i) that differences in ε^114/110^Cd of approximately 3 to 6 ε-units can be produced by a 100 to 200 ppm increase in ion beam intensity at either mass 111 or 114, and (ii) that changes in Cd isotope compositions will exhibit a linear correlation with the magnitude of the interferences (see ESI†). Our data therefore indicate that the observed analytical artefacts are not due to spectral interferences, because the Cd isotope compositions of refluxed NIST Cd samples do not decrease linearly with dilution factor (Fig. 3).

The results obtained for such refluxed samples, however, cast further light on the processes responsible for the poor analytical results. The residual organic material, when only partially oxidised, has a clear impact on the instrumental sensitivity that is achieved for Cd (Fig. 3). A simple explanation of this would be that organic material alters the behaviour of the membrane of the desolvation unit that is used for sample introduction. However, as the reduction in sensitivity is accompanied by changes in the isotopic composition, we suggest that this is not the case and that a better explanation may be that the introduction of organic residue changes the conditions within the plasma and/or the plasma interface. Given that the organic matrix material has such a significant effect on sensitivity, it is conceivable that it can also impact the mass bias behavior to a sufficient extent to produce inaccurate results, despite use of the double spike technique. Indeed, unusual mass bias conditions (as quantified by *f*_Kin_) are observed for about half of the bracketing NIST Cd standards that were analyzed after refluxed samples. Hence, we speculate that the poor data quality is most likely related to anomalies in the absolute mass bias and mass bias behavior that are caused by the presence of organic compounds. We demonstrate this to be feasible by modelling the effects of changing the mass dependence of the instrumental mass fractionation. To this end, we use the general power law (GPL)5
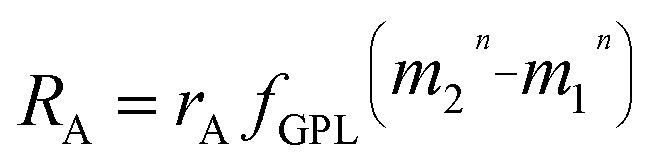
where *R*_A_ is the mass bias corrected or assumed true isotope ratio corrected for instrumental mass bias, *r*_A_ is the same isotope ratio but fractionated relative to the true value due to instrumental mass fractionation, *m*_*i*_ is the atomic mass of isotope *i*, and *n* controls the type or extent of mass dependence (*n* = 1 for the power law, *n* = –1 for the equilibrium law, and *n* → 0 for the exponential/kinetic law) that is applied to correct for the instrumental mass fractionation.^23^

Under normal circumstances, our data reduction applies *n* ≈ –0.1,^23^ but other values may be more appropriate for samples that are contaminated with particular organic compounds. To test this, we recalculated the instrumental mass fractionation-corrected (or assumed true) isotope compositions of refluxed NIST Cd samples using a range of *n* values, relative to analyses of the bracketing standard, which were corrected with *n* = –0.1. For all refluxed NIST Cd samples, there is a linear relationship with negative gradient between *n* and ε^114/110^Cd (see ESI†). This implies that the inaccurate ε^114/110^Cd data for refluxed NIST Cd samples can, in principle, be corrected to accurate results simply by adjusting the *n* to a more appropriate value. In detail, the modelling shows that *n* values of between 0.2 and 0.6 are needed for such a correction (see ESI†). This finding has two main implications. Firstly, our results indicate that the presence of organic compounds generates a mass dependence of mass fractionation that is intermediate between the kinetic/exponential and power laws (*n* ≈ 0.2 to 0.6 for refluxed NIST Cd samples) whilst it is normally intermediate between the kinetic/exponential and equilibrium laws (*e.g.*, *n* ≈ –0.1 for unprocessed NIST Cd). This suggests that the organic material induces a significant change in the processes that are responsible for the instrumental mass bias. Secondly, the range of calculated *n* values implies that the induced changes in mass bias and mass dependence are either not constant, or that the samples differ in their matrix content. Neither of these explanations can be ruled out at present. As changes in the mass dependence of isotope fractionation can produce both positive or negative artefacts, this process may also explain the deviations towards lighter isotope compositions seen by Gault-Ringold and Stirling.^13^

The sources of instrumental mass bias in MC-ICP-MS are not well understood, but the majority of the relevant processes are generally thought to occur either in the plasma or the interface region of the instruments. In the plasma, the radial spread of ions is mass dependent so the portion of the sample that passes through the aperture in the sample cone has a heavier isotope composition than the original sample.^26,27^ In addition the ionisation environment of the plasma can be altered by the presence of matrix, so the distribution of isotopes in the plasma can also change. This has the potential to have an impact on both instrumental sensitivity and isotope ratios, as under normal running conditions the plasma sampling depth (*i.e.* distance between the load coil and sampler cone) is kept constant.^26^ Between the sample and skimmer cones, ions gain kinetic energy by supersonic expansion. Lighter ions do not gain as much kinetic energy as heavier ions, and are therefore more likely to be deflected away from the beam axis when collisions with argon neutrals occur.^27^ Specifically for Cd, Kivel *et al.* ^28^ showed that the Cd exiting the skimmer cone has a heavier isotope composition than the original sample, and that the outer part of the ion beam before the first lens features a lighter Cd isotope composition than the central region. Therefore, we speculate that the presence of organic compounds, which are produced by partial oxidation of organic material leached from Eichrom TRU resin, may change the distribution of isotopes in the plasma and thus alter both the extent and mass dependence of the instrumental mass bias.

## 4. Conclusion

This study provides further evidence that organic resin residues can have a significant detrimental impact on the reproducibility and accuracy of Cd isotope analyses by MC-ICP-MS. These organic residues are eluted from Eichrom TRU resin during Cd purification by extraction chromatography and do not evaporate, even when heated to high temperatures. When left untreated, the organic residue can cause the Cd isotope composition of samples to be isotopically heavier than expected, and this effect is even more severe (with analytical artefacts of up to ∼6.6 ε^114/110^Cd) when samples are refluxed with concentrated nitric acid prior to analysis. In such cases, the instrumental sensitivity for Cd is also reduced by up to ∼40%. Our results indicate that spectral interferences are unlikely to be the cause of these analytical artefacts. Rather, they are probably related to changes in the extent and mass dependence of the instrumental mass bias, which are induced by the presence of the resin-derived organic material. These analytical problems for Cd isotope measurements are circumvented by performing a quick and easy liquid–liquid extraction step using heptane, which removes resin-derived organic compounds from sample solutions. Given the efficiency of this method it should be particularly appropriate for analyses of samples with low Cd contents.

It is common knowledge that traces of organic material are leached from the resins that are commonly used in isotope geochemistry for sample purification by ion exchange and extraction chromatography. In many cases, this leaching is not problematic and has no impact on data quality. However, as ever-more ambitious analytical targets are addressed, more analytical problems related to resin-derived organics may become apparent and knock-on effects may arise when the problematic resin is not used in the final purification stage. Although not yet tested on organic compounds eluted from resins other than Eichrom TRU, the principles of our liquid–liquid extraction technique should be applicable to and may solve problems associated with other resin types and isotopic analyses of other elements.
