# Supplementary material for: Improvements in Cd stable isotope analysis achieved through use of liquid–liquid extraction to remove organic residues from Cd separates obtained by extraction chromatography
Source: J Anal At Spectrom. 2015 Aug 18;31(1):319–27. doi: 10.1039/c5ja00115c (PMC4869401; doi:10.1039/c5ja00115c)
Supplement: Supplementary file 1 [file JA-031-C5JA00115C-s001.pdf]

Electronic Supplementary Material (ESI) for Journal of Analytical Atomic Spectrometry.
This journal is © The Royal Society of Chemistry 2015

Supplementary Information to:

Improvements in Cd stable isotope analysis achieved through use of liquid-liquid extraction to remove organic residues from Cd separates obtained by extraction chromatography

Katy Murphy[1]*, Mark Rehkämper[1], Katharina Kreissig[1], Barry Coles[1], Tina van de Flierdt[1]

[1]Department of Earth Science and Engineering, Imperial College London,
London SW7 2AZ, UK
*Corresponding author. Email: k.murphy12@imperial.ac.uk. Tel. +44 2075947140

S1 Complete Cd isotope data set for NIST 3108 Cd and Nod-A-1 samples. See main text for description of sample treatment methods. The reduction in sensitivity was not calculated for all samples, but it is appropriate to assume that the reduction was negligible unless stated.

Sample	Type	Mass Cd (ng)	Treatment	$\varepsilon^{114/110}$Cd	2SE (internal)	2SD (external)	Number of bracketing standards	Included in Table 2?	Reduction in sensitivity (%)	Comments
1	NIST Cd	60	Untreated	-0.14	1.35	0.83	3	No		
2	NIST Cd	60	Untreated	0.49	0.67	0.42	4	Yes		
3	NIST Cd	60	Untreated	0.06	0.77	0.65	5	Yes		
4	NIST Cd	60	Untreated	-0.44	0.80	1.00	9	Yes		
5	NIST Cd	60	Untreated	0.25	0.84	0.43	5	Yes		
6	NIST Cd	60	Untreated	-0.55	0.82	0.59	6	Yes		
7	NIST Cd	60	Untreated	-0.31	0.58	0.62	34	Yes		
8	NIST Cd	30 + 30	Refluxed	3.09	0.85	0.65	68	No	39	
9	NIST Cd	30	Refluxed	3.48	1.13	0.85	8	No	33	
10	NIST Cd	59	Refluxed	1.41	1.25	1.51	5	No	31	
11	NIST Cd	60	Refluxed	0.95	0.98	1.18	6	No	21	
12	NIST Cd	59	Refluxed	7.23	1.36	2.03	5	No	42	
13	NIST Cd	59	Refluxed	4.00	0.94	1.01	10	No	28	
14	NIST Cd	60	Refluxed	5.08	0.87	0.62	11	Yes	35	
15	NIST Cd	60	Refluxed	6.64	0.73	0.24	6	Yes	36	
16	NIST Cd	59	Refluxed	3.79	1.15	0.77	6	Yes	34	Internal precision not unusual for session
17	NIST Cd	60	Refluxed	2.73	0.65	0.62	34	Yes	36	
18	NIST Cd	79	Refluxed	1.17	0.98	0.78	6	No	19	
19	NIST Cd	98	Refluxed	1.05	0.98	0.67	6	No	17	
20	NIST Cd	118	Refluxed	1.65	0.70	0.94	8	No	10	

S1 continued

Sample	Type	Mass Cd (ng)	Treatment	$\varepsilon^{114/110}$Cd	2SE (internal)	2SD (external)	No. bracketing standards	Included in Table 2?	Reduction in sensitivity (%)	Comments
21	NIST Cd	118	Refluxed	0.57	1.00	0.75	8	No	18	
22	NIST Cd	118	Refluxed	0.73	1.18	0.59	6	No	13	
23	NIST Cd	176	Refluxed	-0.51	0.84	0.66	7	No	5	
24	NIST Cd	176	Refluxed	0.23	0.83	0.63	6	No	-1	
25	NIST Cd	178	Refluxed	0.25	0.90	0.45	8	No	5	
26	NIST Cd	235	Refluxed	0.41	0.89	0.59	9	No	-6	
27	NIST Cd	235	Refluxed	0.09	1.18	0.87	6	No	2	
28	NIST Cd	60	Refluxed (overnight only)	3.85	0.91	0.80	6	No		
29	NIST Cd	60	Extracted (once)	0.44	1.06	0.48	6	No		
30	NIST Cd	60	Extracted	1.12	0.77	1.86	10	No		Run after several refluxed samples, instrument possibly unstable
31	NIST Cd	60	Extracted	-0.31	1.10	0.69	6	No		
32	NIST Cd	60	Extracted	-0.21	1.17	0.54	7	Yes		Internal precision not unusual for session
33	NIST Cd	60	Extracted	0.33	0.95	0.51	8	Yes		
34	NIST Cd	60	Extracted	-0.34	0.88	0.74	8	Yes		
35	NIST Cd	60	Extracted	-0.19	0.46	0.62	34	Yes		
36	NIST Cd	60	Extracted	0.00	0.69	0.62	34	Yes		
37	NIST Cd	60	Extracted	0.21	0.42	0.62	7	Yes		
38	NIST Cd	60	Extracted (once), refluxed	-0.19	0.56	0.63	6	No		
39	NIST Cd	60	Extracted and refluxed	0.07	0.52	1.15	5	No		
40	NIST Cd	60	Extracted and refluxed	0.07	0.46	0.46	5	Yes		
41	NIST Cd	60	Extracted and refluxed	-0.51	0.59	0.74	7	Yes		
42	NIST Cd	60	Extracted and refluxed	0.14	0.70	0.43	6	Yes		
43	NIST Cd	60	Extracted and refluxed	0.19	0.63	0.34	8	Yes		
44	NIST Cd	60	Extracted and refluxed	-0.14	0.57	0.76	9	Yes		

S1 continued

Sample	Type	Mass Cd (ng)	Treatment	$\varepsilon^{114/110}$Cd	2SE (internal)	2SD (external)	Number of bracketing standards	Included in Table 2?	Reduction in sensitivity (%)	Comments
45	NIST Cd	60	Extracted and refluxed	0.22	0.67	0.47	9	Yes		
46	NIST Cd	60	Extracted and refluxed	-0.47	0.53	0.81	6	Yes		
47	NIST Cd	60	Extracted and refluxed	0.48	0.44	0.60	7	Yes		
48	NIST Cd	60	Extracted and refluxed	0.32	0.57	0.67	7	Yes		
49	NIST Cd	60	Refluxed and extracted	2.69	0.62	0.37	6	Yes		
50	NIST Cd	60	Refluxed and extracted	2.85	0.62	0.62	34	Yes		
51	NIST Cd	60	Refluxed and extracted	3.42	0.60	0.62	34	Yes		
52	Nod-A-1	60	Untreated	3.73	0.69	0.66	6	Yes		
53	Nod-A-1	60	Untreated	1.34	0.71	0.76	9	Yes		
54	Nod-A-1	60	Untreated	3.01	1.57	0.96	7	No	87	Low beam intensity
55	Nod-A-1	120	Untreated	1.70	1.06	0.52	8	No		
56	Nod-A-1	60	Refluxed	6.36	0.86	0.62	8	Yes		
57	Nod-A-1	60	Refluxed	2.20	0.98	0.72	18	Yes		
58	Nod-A-1	120	Refluxed	1.80	0.83	0.52	6	No		
59	Nod-A-1	60	Extracted	1.98	0.55	0.56	9	Yes		
60	Nod-A-1	60	Extracted	1.36	0.62	0.87	6	Yes		
61	Nod-A-1	60	Extracted	1.51	0.90	0.76	8	Yes		
62	Nod-A-1	60	Extracted	1.90	0.69	0.70	28	Yes		
63	Nod-A-1	60	Extracted and refluxed	1.97	0.70	1.24	9	Yes		
64	Nod-A-1	60	Extracted and refluxed	2.37	0.81	0.87	8	Yes		
65	Nod-A-1	60	Refluxed and extracted	4.04	1.08	0.72	6	Yes	58	Low beam intensity
66	Nod-A-1	60	Refluxed and extracted	3.16	0.80	0.53	6	Yes		
67	Nod-A-1	120	Refluxed and extracted	2.24	0.74	0.52	8	No		

S2 Further information on untreated Nod-A-1 analyses

Of the three samples of untreated Nod-A-1 that were analyzed, only one produced a reasonable result, of $\varepsilon^{114/110}Cd = 1.3 \pm 0.8$, which is in good agreement with literature values (Table 2). The second sample yielded an isotopic composition that was significantly heavier than literature values ($\varepsilon^{114/110}Cd = 3.7 \pm 0.7$). This analysis was also compromised by a 2.2 ε unit drop in the data obtained for subsequent measurements of the bracketing standard, so these analyses of the standard were excluded from the calculation of the sample isotope composition. This 'drop' occurred between the first and second analyses of the standard solution after the Nod-A-1 sample, and was accompanied by large changes in f_{Kin} (Fig. 2). The instrumental sensitivity obtained during the analysis of the third untreated Nod-A-1 sample was just 14% of the expected level. Incomplete dissolution of the Cd fraction in 0.1 M HNO_3 is unlikely to be responsible for this dramatic reduction in sensitivity. A more plausible explanation is that the organic resin residue disrupted the normal behavior of the instrument and/or sample introduction system.

S3 Modeling of spectral interferences

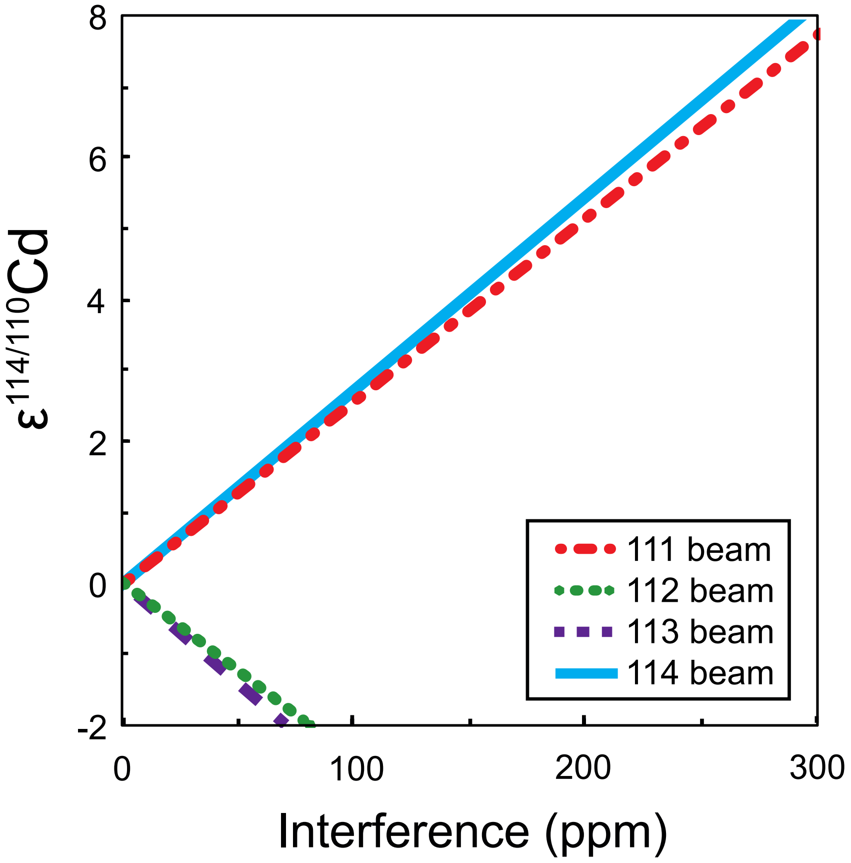

The modeling shows that it is possible that a spectral interference on one ion beam can alter the mass bias corrected $\varepsilon^{114/110}$Cd value. These experiments were conducting using the ion beam intensity data from a single measurement of a NIST SRM 3108 Cd standard solution (with $\varepsilon^{114/110}$Cd = 0) that was doped with the Cd double spike (to S/N ≈ 1) to obtain a total Cd concentration of 60 ng/ml. In the modeling, the ion beam intensity data were manipulated to simulate an interference on a single ion beam. This was repeated for a range of interference magnitudes, and on each ion beam. The Cd isotope compositions were subsequently calculated using the normal double spike data reduction spreadsheet, as an offset from the original (unaltered) Cd isotope composition.

S4 Modeling the effects of changing the mass dependence (*n* factor) of the General Power Law that is used for correction of the instrumental mass fractionation

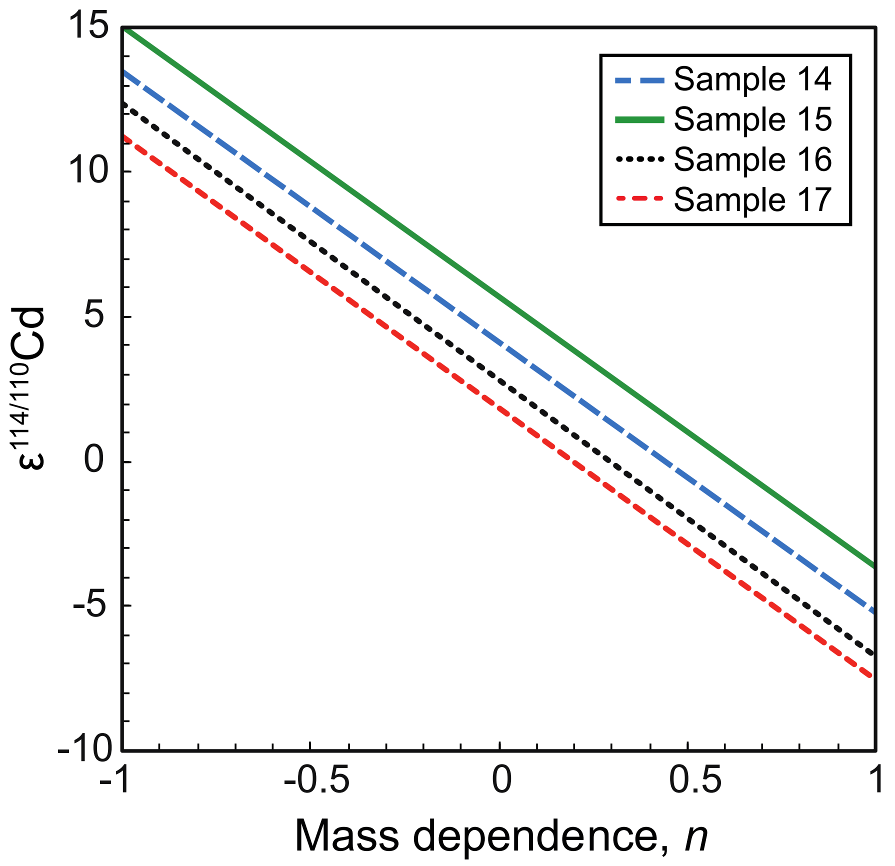

The mass bias corrected isotope composition of a sample was recalculated for values of *n* (which describes the mass dependence of the instrumental mass fractionation correction that is applied using the General Power Law) between -1 and 1 for four refluxed NIST Cd samples with 60 ng of total Cd. Using our normal value of $n = -0.1$, these samples yielded $\varepsilon^{114/110}$Cd values of between +2.7 and +6.4. Notably, each sample can be corrected to the reference value of $\varepsilon^{114/110}$Cd = 0 using *n* values of 0.2 to 0.6. This is a reasonable range of values as they are intermediate between the kinetic/exponential and power laws. Sample numbers refer to the data presented in the Table of S1.
